# Integrating gene expression and clinical data to identify drug repurposing candidates for hyperlipidemia and hypertension

**DOI:** 10.1038/s41467-021-27751-1

**Published:** 2022-01-10

**Authors:** Patrick Wu, QiPing Feng, Vern Eric Kerchberger, Scott D. Nelson, Qingxia Chen, Bingshan Li, Todd L. Edwards, Nancy J. Cox, Elizabeth J. Phillips, C. Michael Stein, Dan M. Roden, Joshua C. Denny, Wei-Qi Wei

**Affiliations:** 1 Department of Biomedical Informatics, Vanderbilt University Medical Center, Nashville, TN USA; 2 Medical Scientist Training Program, Vanderbilt University School of Medicine, Nashville, TN USA; 3 Division of Clinical Pharmacology, Department of Medicine, Vanderbilt University Medical Center, Nashville, TN USA; 4 Division of Allergy, Pulmonary and Critical Care Medicine, Department of Medicine, Vanderbilt University Medical Center, Nashville, TN USA; 5 Department of Biostatistics, Vanderbilt University School of Medicine, Nashville, TN USA; 6 Department of Molecular Physiology and Biophysics, Vanderbilt University School of Medicine, Nashville, TN USA; 7 Vanderbilt Genetics Institute, Vanderbilt University Medical Center, Nashville, TN USA; 8 Biomedical Laboratory Research and Development, Tennessee Valley Healthcare System (626)/Vanderbilt University, Nashville, TN USA; 9 Division of Epidemiology, Department of Medicine, Vanderbilt University Medical Center, Nashville, TN USA; 10 Institute for Medicine and Public Health, Vanderbilt University Medical Center, Nashville, TN USA; 11 Department of Medicine, Vanderbilt University Medical Center, Nashville, TN USA; 12 Division of Infectious Diseases, Department of Medicine, Vanderbilt University Medical Center, Nashville, TN USA; 13 Department of Pharmacology, Vanderbilt University School of Medicine, Nashville, TN USA; 14 Department of Pathology, Microbiology and Immunology, Vanderbilt University Medical Center, Nashville, TN USA; 15 Institute for Infectious Diseases and Immunology, Murdoch University, Murdoch, Western Australia Australia; 16 All of Us Research Program, National Institutes of Health, Bethesda, MD USA; 17 National Human Genome Research Institute, National Institutes of Health, Bethesda, MD USA

**Keywords:** Virtual drug screening, Gene expression, Data integration, High-throughput screening, Software

## Abstract

Discovering novel uses for existing drugs, through drug repurposing, can reduce the time, costs, and risk of failure associated with new drug development. However, prioritizing drug repurposing candidates for downstream studies remains challenging. Here, we present a high-throughput approach to identify and validate drug repurposing candidates. This approach integrates human gene expression, drug perturbation, and clinical data from publicly available resources. We apply this approach to find drug repurposing candidates for two diseases, hyperlipidemia and hypertension. We screen >21,000 compounds and replicate ten approved drugs. We also identify 25 (seven for hyperlipidemia, eighteen for hypertension) drugs approved for other indications with therapeutic effects on clinically relevant biomarkers. For five of these drugs, the therapeutic effects are replicated in the All of Us Research Program database. We anticipate our approach will enable researchers to integrate multiple publicly available datasets to identify high priority drug repurposing opportunities for human diseases.

## Abstract (web summary)

Prioritizing drug repurposing candidates for downstream studies remains challenging. Here, the authors present a high-throughput approach to identify and validate drug repurposing candidates, integrating human gene expression, drug perturbation, and clinical data from publicly available resources.

## Introduction

Developing a new drug is expensive, often fails, and takes a long time. Drug repurposing aims to address these issues by finding new indications for existing drugs^1^. Repurposing existing drugs can decrease the cost and shorten the duration of drug development because many of the preclinical and safety studies have already been completed. Drug repurposing can also improve the success rate of drug development because existing drugs often have well-characterized safety profiles. Examples of successfully repurposed drugs include rituximab for rheumatoid arthritis^2^ and sildenafil for erectile dysfunction^3^. However, there have also been many drug repurposing candidates that have failed in clinical trial testing due to lack of efficacy^1,4^.

To address this challenge, researchers have developed high-throughput approaches leveraging human genetic data to identify effective repurposing candidates^5,6^. These methods are supported by the finding that drugs are more likely to pass clinical trials if their targets overlap with hits from human genetics studies^7,8^. An emerging approach using human genetic data to identify repurposing candidates is based on the hypothesis that a drug that reverses the molecular state of disease would also be an effective treatment for the disease^9,10^. To represent the molecular state of a disease, this approach calculates a gene expression signature using summary statistics from a genome-wide association study (GWAS) for the disease^11,12^. The disease gene expression signature is then used to search for drugs that reverse the disease-associated gene expression changes^13,14^. While these studies generate many repurposing signals, it remains a challenge to determine which of the repurposing candidates have the highest likelihood of passing clinical trials with commonly used validation methods (Supplementary Fig. 1).

To validate drug repurposing candidates, researchers commonly use animal models and in vitro assays, but these methods have two major limitations. First, these validation tools are sub-optimal representations of human disease, so evidence generated using these tools often serve as unreliable predictors for drug response in humans. There are instances of repurposing candidates that were effective in animal models^10^ but subsequently failed to work in humans^4,15^. A second limitation is that using these methods to test most of the repurposing candidates identified from human genetic data is both time- and cost-prohibitive (e.g., a recent study identified 210 drug repurposing candidates for hypertension^14^), so researchers can only test a handful of repurposing candidates. Consequently, among the repurposing candidates not tested, there may be a drug that is effective at treating the disease of interest. In contrast, generating reliable evidence to predict drug response in humans for many repurposing candidates can be done quickly and cost-effectively using clinical data from electronic health records (EHRs)^16,17^.

**Fig. 1:**
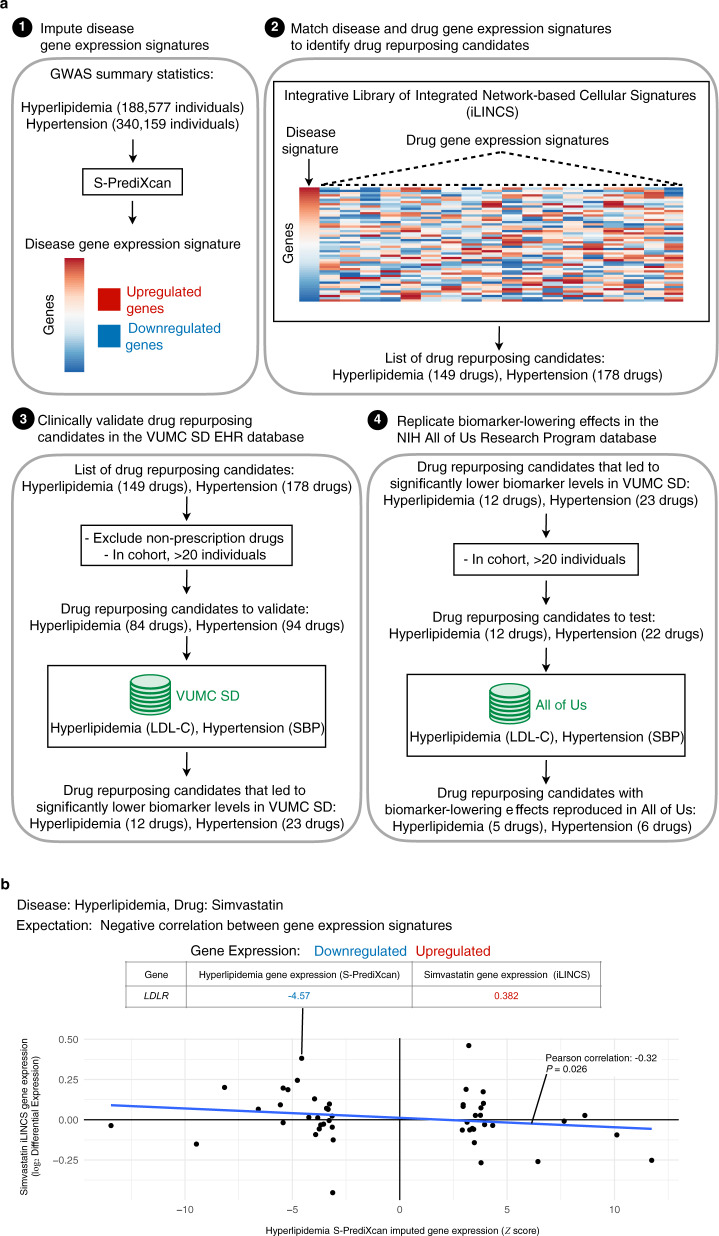
Study design and workflow. **a** (1) For each disease, disease-associated gene expression changes were imputed using each disease’s GWAS summary statistics and S-PrediXcan. Using the list of imputed disease-associated gene expression changes, the top up- and downregulated genes were used to compute a disease gene expression signature. (2) The disease gene expression signature was then uploaded to the drug perturbation platform, iLINCS. From iLINCS, an initial list of drug repurposing candidates was obtained; drugs in this list induced perturbations that reversed the disease gene expression signature (see **b**). (3) A subset of the iLINCS drug repurposing candidates was clinically validated in the VUMC SD EHR database (Fig. 2). (4) Drugs with significant biomarker-lowering effects in the VUMC SD were chosen for replication studies in the NIH All of Us Research Program database. **b** Example of disease and drug-gene expression signature matching (second step in **a**). Each point represents one gene. Since simvastatin is a known lipid-lowering drug, the simvastatin induced gene expression signature was predicted to reverse the S-PrediXcan imputed gene expression signature for hyperlipidemia, i.e., the signatures were expected to have an inverse relationship. This inverse relationship is indicated by the blue line, which shows a negative correlation (Pearson correlation coefficient and two-tailed test *P*-value) between the S-PrediXcan imputed gene expression signature for hyperlipidemia (horizontal axis) and the iLINCS gene expression signature for simvastatin (vertical axis). As expected, the *LDLR* gene was downregulated in individuals with hyperlipidemia and upregulated in simvastatin perturbation experiments. GWAS genome-wide association study, iLINCS Integrative Library of Integrated Network-based Cellular Signatures, EHR electronic health record, LDL-C low-density lipoprotein cholesterol, SBP systolic blood pressure, VUMC Vanderbilt University Medical Center, SD Synthetic Derivative, NIH National Institutes of Health.

Here, we describe a proof-of-concept approach integrating imputed human disease gene expression signatures, drug perturbation data, and clinical EHR data to identify and validate repurposing candidates. The four major steps of this approach are (1) imputing human disease gene expression signatures using S-PrediXcan^11,12^ and GWAS summary statistics, (2) searching for drugs that reverse the disease gene expression signatures in drug perturbation databases using the Integrative Library of Integrated Network-based Cellular Signatures (iLINCS) platform^18,19^, (3) validating iLINCS repurposing candidates, using clinical data stored in the Synthetic Derivative (SD), the de-identified EHR database at Vanderbilt University Medical Center (VUMC), and (4) replicating repurposing candidate signals using clinical data stored in the National Institutes of Health (NIH) All of Us Research Program database (Fig. 1a)^20,21^. We applied this approach to find repurposing candidates for two diseases, hyperlipidemia and hypertension. We chose these diseases to test this proof-of-concept approach because they have several known US Food and Drug Administration (FDA)-approved drugs and robust biomarkers to measure drug efficacy. The data used in this study, except for individual-level clinical data in the VUMC SD, are all stored in publicly available databases. We have also made the software tools available in open source for researchers to apply this drug repurposing approach for their diseases of interest.

## Results

### Using gene expression to find drug repurposing candidates

We developed a novel approach integrating disease gene expression signatures, drug perturbation data, and clinical data, to identify and validate drug repurposing candidates. To compute the gene expression signature for each disease, we searched a public database with disease-associated gene expression changes^22,23^. These disease-associated gene expression changes were imputed using each disease’s GWAS summary statistics^24,25^ and S-PrediXcan^11,12^. For both disease signatures, the direction of gene expression changes for known disease-associated genes was concordant with existing knowledge. For example, in the gene expression signature for hyperlipidemia, *PCSK9*^26^ was upregulated and *LDLR*^27^ was downregulated (Supplementary Data 1), as expected. In the gene expression signature for hypertension, *ADRB1* and *ACE* were both upregulated (Supplementary Data 1), as expected. We then uploaded each disease’s gene expression signatures to iLINCS (Supplementary Data 2–5). In iLINCS, we found 149 and 178 drugs with perturbation signatures that reversed the disease gene expression signatures for hyperlipidemia and hypertension, respectively (Fig. 1b and Supplementary Data 6 and 7).

### Validating drug repurposing candidates with clinical data

**Fig. 2:**
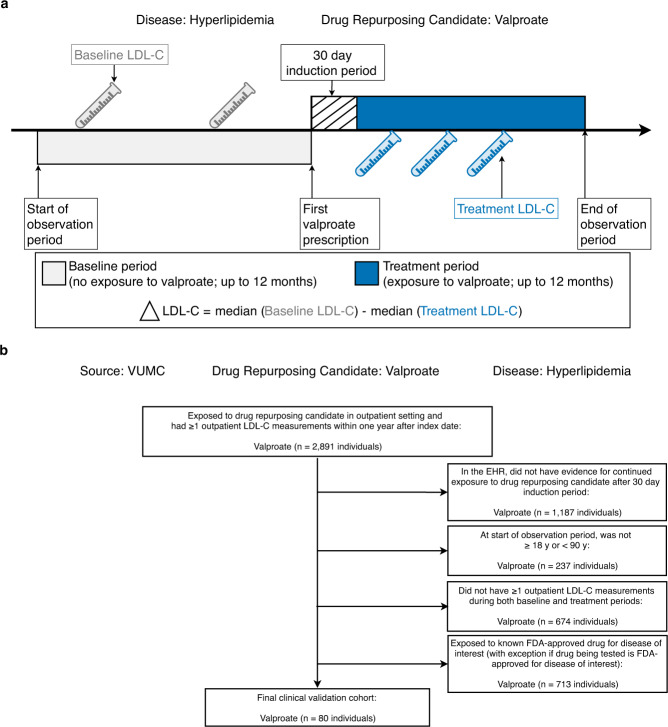
Clinical validation study design. **a** Example of SCCS design used for clinical validation studies in EHRs. The target disease is hyperlipidemia, and the drug repurposing candidate is valproate. The outcome is the change in median LDL-C from baseline after exposure to valproate. For each drug repurposing candidate analysis, individuals were generally from different time periods. **b** Example clinical validation study cohort selection flow chart. See Supplementary Data 8 for cohort selection numbers. VUMC Vanderbilt University Medical Center, LDL-C low-density lipoprotein cholesterol, EHRs electronic health records, SCCS self-controlled case series.

Next, we performed clinical validation studies to test the ability of the signature-based approach to rediscover known approved drugs and to identify new candidate drugs not currently approved for treating the diseases of interest. We performed these validation studies using clinical data stored in the VUMC SD^28^, which contained de-identified EHRs for >3.2 million individuals at the time of the study. We tested prescription drugs with at least twenty individuals in the clinical validation cohort (Supplementary Fig. 2) using a self-controlled case series (SCCS) study design^29^ (Fig. 2a). Consider, for example, the clinical validation study of valproate as a repurposing candidate for hyperlipidemia. In this experiment, we measured the change in low-density lipoprotein cholesterol (LDL-C) levels due to valproate exposure in the outpatient setting. For each individual, we defined an observation period composed of two parts, a baseline period (before valproate exposure) and a treatment period (after valproate exposure). The baseline and treatment periods were divided by the index date, defined as the first date each individual was exposed to valproate. We calculated the outpatient median LDL-C measurements for both baseline and treatment periods, respectively. To adjust for potential confounding by indication, we excluded individuals who were exposed to any known FDA-approved lipid-lowering drugs during the observation period (Fig. 2b). To determine whether individuals experienced statistically significant reductions in LDL-C after valproate exposure, we used a linear mixed model.

**Fig. 3:**
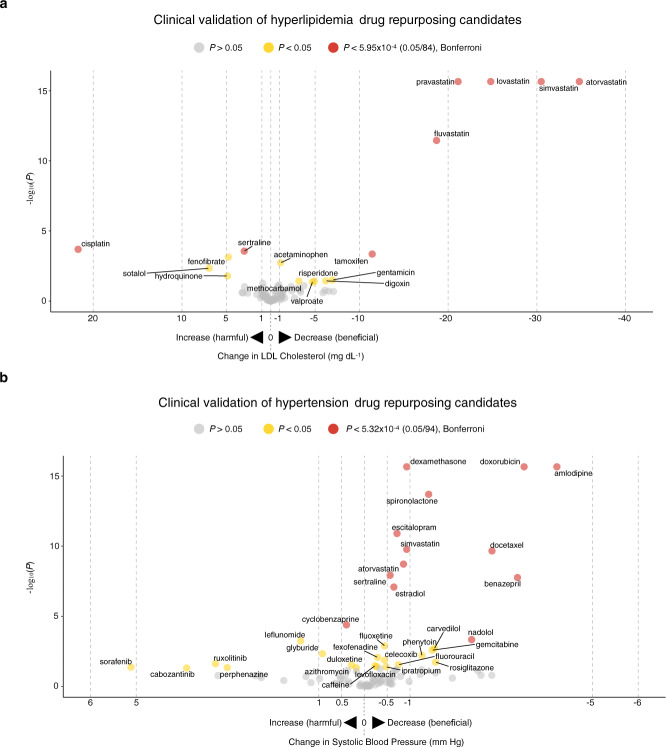
Clinical validation study results for drug repurposing candidates in VUMC EHR database. **a** Results from clinical validation studies for hyperlipidemia repurposing candidates. **b** Results from clinical validation studies for hypertension repurposing candidates. For hyperlipidemia, the biomarker was LDL-C; for hypertension, the biomarker was SBP. On the vertical axis, −log_10_(*P*) is plotted; on the horizontal axis, change in biomarker measurements from baseline after drug exposure is plotted. Each point indicates one drug. On the horizontal axis, drugs plotted to the right of 0 indicate that individuals experienced reductions in biomarker measurements after drug exposure. Drugs plotted to the left of 0 indicate that individuals had elevated biomarker measurements after drug exposure. Two-tailed *P* values were calculated using linear mixed models. Drugs with *P* > 0.05 are in gray, *P* < 0.05 are in yellow, and *P* values that passed Bonferroni significance (to correct for multiple comparisons) are in red. Drugs with *P* < 2.2 × 10^−16^ were transformed to 2.2 × 10^−16^ for visualization purposes. EHR electronic health record, VUMC Vanderbilt University Medical Center, SD Synthetic Derivative, LDL-C low-density lipoprotein cholesterol, SBP systolic blood pressure.

For the hyperlipidemia clinical validation study, we quantified the effects of 84 drugs on LDL-C levels. In this analysis, we removed individuals who were exposed to other known FDA-approved lipid-lowering drugs during the observation period (Fig. 2b and Supplementary Data 8). The sociodemographic characteristics and comorbidities of the individuals studied are shown in Supplementary Data 9–11, and LDL-C measurements during both baseline and treatment periods can be found in Supplementary Data 12. Out of the 84 drugs tested, 12 lowered LDL-C with *P* < 0.05 (Fig. 3a and Supplementary Data 13). Five of the repurposing signals were statins, the most commonly used FDA-approved lipid-lowering drugs: fluvastatin (LDL-C mg dL^−1^, point estimate [95% confidence interval (CI)] = −18.7 [−23.5, −13.9], *P* = 3.50 × 10^−12^), pravastatin (−21.1 [−22.4, −19.9], *P* < 2.20 × 10^−16^), lovastatin (−24.8 [−26.9, −22.8], *P* < 2.20 × 10^−16^), simvastatin (−30.5 [−31.4, −29.6], *P* < 2.20 × 10^−16^), and atorvastatin (−34.8 [−35.7, −33.9], *P* < 2.20 × 10^−16^). The other seven signals were drugs FDA-approved for other diseases: acetaminophen (LDL-C mg dL^−1^, point estimate [95% CI] = −1.12 [−1.83, −0.41], *P* = 1.85 × 10^−3^), methocarbamol (−3.18 [−6.16, −0.20], *P* = 0.04), valproate (−4.71 [−9.21, −0.19], *P* = 0.04), risperidone (−4.93 [−9.54, −0.32], *P* = 0.04), digoxin (−6.21 [−12.0, −0.45], *P* = 0.04), gentamicin (−6.97 [−13.2, −0.74], *P* = 0.03), and tamoxifen (−11.4 [−17.6, −5.32], *P* = 4.48 × 10^−4^). Among the 12 drugs, 6 lowered LDL-C with *P* values crossing the Bonferroni threshold (0.05/84 = 5.95 × 10^−4^), 5 of which were known drugs approved for treating hyperlipidemia, and one approved for treating other diseases (Table 1).

**Table 1:** Summary of clinical validation study findings.

Source			Hyperlipidemia	Hypertension
Vanderbilt				
	Drug repurposing candidates tested		84	94
	Therapeutic effect & *P* < 0.05		12	23
		Drugs approved for target disease	5	5
		Drugs approved for other diseases	7	18
	Therapeutic effect & *P* < Bonferroni		6	12
		Drugs approved for target disease	5	4
		Drugs approved for other diseases	1	8
All of Us				
	Drug repurposing candidates tested		12	22
	Therapeutic effect & *P* < 0.05		5	6
		Drugs approved for target disease	4	2
		Drugs approved for other diseases	1	4

Therapeutic effect means that individuals experienced reductions in biomarker measurements (LDL-C for hyperlipidemia; SBP for hypertension) after exposure to the drug repurposing candidate.

Two-tailed *P* values were calculated using linear mixed models.

For the clinical validation studies at Vanderbilt, we report both the number of drugs with *P* < 0.05 and *P* values that pass Bonferroni significance to correct for multiple comparisons. For the replication studies in All of Us, we report the number of drugs with *P* < 0.05.

*LDL-C* low-density lipoprotein cholesterol, *SBP* systolic blood pressure.

For the hypertension clinical validation study, we quantified the effects of 94 drugs on systolic blood pressure (SBP). In this analysis, we removed individuals who were exposed to other known FDA-approved antihypertensive drugs during the observation period (Fig. 2b and Supplementary Data 8). The sociodemographic characteristics and comorbidities of the individuals studied are shown in Supplementary Data 9–11, and SBP measurements during both baseline and treatment periods can be found in Supplementary Data 12. Out of the 94 drugs tested, 23 lowered SBP with *P* < 0.05 (Fig. 3b and Supplementary Data 13). Five of the repurposing signals were known FDA-approved antihypertensive drugs: spironolactone (SBP mm Hg, point estimate [95% CI] = −1.41 [−1.76, −1.06], *P* = 2.02 × 10^−14^), carvedilol (−1.54 [−2.50, −0.58], *P* = 1.92 × 10^−3^), nadolol (−2.35 [−3.66, −1.04], *P* = 4.63 × 10^−4^), benazepril (−3.35 [−4.51, −2.19], *P* = 1.74 × 10^−8^), and amlodipine (−4.22 [−4.67, −3.77], *P* < 2.20 × 10^−16^). The other eighteen signals were drugs FDA-approved for other diseases: caffeine (SBP mm Hg, point estimate [95% CI] = (−0.23 [−0.45, −0.01], *P* = 0.03), levofloxacin (−0.27 [−0.52, −0.02], *P* = 0.04), fexofenadine (−0.29 [−0.51, −0.07], *P* = 8.61 × 10^−3^), fluoxetine (−0.44 [−0.71, −0.17], *P* = 1.28 × 10^−3^), celecoxib (−0.44 [−0.79, −0.09], *P* = 0.01), ipratropium (−0.48 [−0.93, −0.03], *P* = 0.04), sertraline (−0.56 [−0.76, −0.36], *P* = 1.20 × 10^−8^), estradiol (−0.65 [−0.89, −0.41], *P* = 8.26 × 10^−8^), escitalopram (−0.71 [−0.93, −0.49], *P* = 1.26 × 10^−11^), fluorouracil (−0.75 [−1.42, −0.08], *P* = 0.03), atorvastatin (−0.86 [−1.13, −0.59], *P* = 1.91 × 10^−9^), simvastatin (−0.93 [−1.22, −0.64], *P* = 1.69 × 10^−10^), dexamethasone (−0.93 [−1.11, −0.75], *P* < 2.20 × 10^−16^), phenytoin (−1.26 [−2.16, −0.36], *P* = 6.22 × 10^−3^), gemcitabine (−1.49 [−2.45, −0.53], *P* = 2.61 × 10^−3^), rosiglitazone (−1.56 [−2.85, −0.27], *P* = 0.02), docetaxel (−2.8 [−3.64, −1.96], *P* = 2.19 × 10^−10^), and doxorubicin (−3.5 [−4.11, −2.89], *P* = 2.20 × 10^−16^). Among the 23 drugs, 12 lowered SBP with *P* values crossing the Bonferroni threshold (0.05/94 = 5.32 × 10^−4^), 4 of which were known drugs approved for treating hypertension and eight drugs indicated for other diseases (Table 1).

### External replication of clinical validation studies

**Fig. 4:**
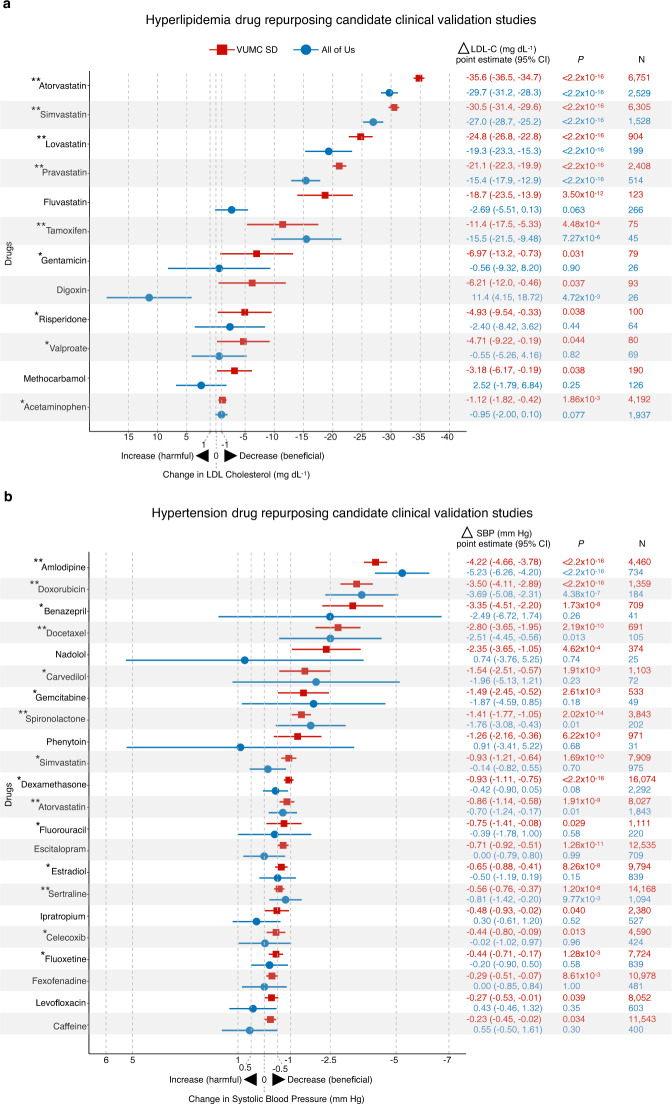
Comparison of clinical validation study treatment effects. Forest plots and treatment effect statistics for clinical validation studies in the VUMC SD (red squares) and NIH All of Us Research Program (blue circles) databases. **a** Hyperlipidemia clinical validation studies. **b** Hypertension clinical validation studies. Plotted are biomarker changes after drug exposure, represented as point estimates (95% CI) from linear mixed models. Drugs with point estimates plotted to the right of 0 indicate that individuals had lower biomarker measurements after drug exposure. Drugs with point estimates plotted to the left of 0 indicate that individuals had elevated biomarker measurements after drug exposure. Two-tailed *P* values were calculated using linear mixed models. “**“ are drugs with replicated treatment effects in the All of Us study at *P* < 0.05. “*“ are drugs that reduced biomarker levels (i.e., negative point estimates) in the All of Us study with *P* > 0.05 and had 95% CIs that overlapped with the 95% CIs from the VUMC SD study. VUMC Vanderbilt University Medical Center, SD Synthetic Derivative, N number of individuals, NIH National Institutes of Health, LDL-C low-density lipoprotein cholesterol, 95% CI 95% confidence interval.

To confirm the VUMC SD clinical validation findings, we performed external replication studies in the NIH All of Us Research Program database^20^. At the time of study, All of Us had EHRs for >236,000 individuals with diverse ancestries. We tested drugs with therapeutic effects (i.e., lowered LDL-C or SBP measurements at *P* < 0.05) in the VUMC SD clinical validation study. The sociodemographic characteristics and comorbidities for both hyperlipidemia and hypertension cohorts can be found in Supplementary Data 9–11. For hyperlipidemia, we tested twelve drugs and found that five lowered LDL-C at *P* < 0.05 (Fig. 4a and Supplementary Data 13). These drugs were pravastatin (LDL-C mg dL^−1^, point estimate [95% CI] = −15.4 [−17.9, −12.9], *P* < 2.20 × 10^−16^), tamoxifen (−15.5 [−21.5, −9.49], *P* = 7.27 × 10^−6^), lovastatin (−19.3 [−23.3, −15.3], *P* < 2.20 × 10^−16^), simvastatin (−27.0 [−28.7, −25.2], *P* < 2.20 × 10^−16^), and atorvastatin (−29.7 [−31.2, −28.3], *P* < 2.20 × 10^−16^). For hypertension, we analyzed 22 drugs and found that six drugs lowered SBP at *P* < 0.05 (Fig. 4b and Supplementary Data 13). These drugs were atorvastatin (SBP mm Hg, point estimate [95% CI] = −0.70 [−1.23, −0.17], *P* = 0.01), sertraline (−0.81 [−1.42, −0.20], *P* = 9.77 × 10^−3^), spironolactone (−1.76 [−3.09, −0.43], *P* = 9.98 × 10^−3^), docetaxel (−2.51 [−4.45, −0.57], *P* = 0.01), doxorubicin (−3.69 [−5.08, −2.30], *P* = 4.38 × 10^−7^), and amlodipine (−5.23 [−6.27, −4.19], *P* < 2.20 × 10^−16^). Though fewer drugs reduced biomarker measurements with *P* < 0.05, most drugs had treatment effects in the expected direction (i.e., negative point estimates) with 95% CIs that overlapped with the 95% CIs from the VUMC SD clinical validation study (Fig. 4).

### Review of evidence to support novel repurposing candidates

We used multiple databases, the literature, and domain-expert review to confirm the treatment effects we observed for drugs indicated for other diseases, i.e., potential repurposing candidates. For hyperlipidemia, we found seven drugs, not approved for treating hyperlipidemia, which had statistically significant LDL-C lowering effects in the VUMC SD clinical validation study. For three of these drugs, we found evidence supporting their LDL-C lowering effects: tamoxifen^30^, digoxin^31^, and valproate^32^. On the other hand, we did not find existing evidence supporting the LDL-C lowering effects for four drugs: gentamicin, risperidone, methocarbamol, and acetaminophen (Table 2 and Supplementary Table 1). Since gentamicin is commonly prescribed in non-systemic forms (e.g., ophthalmic solutions and topical ointments), we conducted a post hoc analysis in the VUMC SD by excluding 23 individuals exposed to non-systemic forms of gentamicin. In this subgroup composed of only 56 individuals exposed to systemic forms of gentamicin, the drug no longer had a statistically significant effect on lowering LDL-C (point estimate [95% CI] = −5.06 [−13.4, 3.25] mg dL^−1^, *P* = 0.24).

**Table 2:** Review of existing evidence to confirm the therapeutic effects for drug repurposing candidates observed in clinical validation studies.

Disease	Drug	Approved indication	Existing evidence supports therapeutic effect
Hyperlipidemia	Tamoxifen	Cancer	Yes^30^
Hyperlipidemia	Gentamicin	Bacterial infections	No
Hyperlipidemia	Digoxin	Arrhythmias	Yes^31^
Hyperlipidemia	Risperidone	Schizophrenia	No^40^
Hyperlipidemia	Valproate	Seizure	Yes^32^
Hyperlipidemia	Methocarbamol	Muscle spasms	No
Hyperlipidemia	Acetaminophen	Pain	No
Hypertension	Caffeine	Fatigue	No
Hypertension	Levofloxacin	Bacterial infections	Yes^33^
Hypertension	Doxorubicin	Cancer	No
Hypertension	Docetaxel	Cancer	Yes^34^
Hypertension	Rosiglitazone	Type 2 Diabetes	Yes^35^
Hypertension	Gemcitabine	Cancer	No
Hypertension	Phenytoin	Seizure	Yes^36^
Hypertension	Simvastatin	Hyperlipidemia	Yes^37^
Hypertension	Dexamethasone	Inflammation	No
Hypertension	Atorvastatin	Hyperlipidemia	Yes^38^
Hypertension	Fluorouracil	Cancer	Yes^34^
Hypertension	Escitalopram	Depression	No^41^
Hypertension	Estradiol	Menopause	Yes^39^
Hypertension	Sertraline	Depression	No^41^
Hypertension	Ipratropium	Asthma	No
Hypertension	Celecoxib	Pain	No
Hypertension	Fluoxetine	Depression	No^41^
Hypertension	Fexofenadine	Allergic Rhinitis	No^42^

See also Supplementary Tables 1 and 2.

For hypertension, we found 18 drugs that were not approved for treating the disease, with statistically significant SBP lowering effects (Table 2 and Supplementary Table 2). For eight of these drugs, we found evidence to support their SBP lowering effects: levofloxacin^33^, docetaxel^34^, rosiglitazone^35^, phenytoin^36^, simvastatin^37^, atorvastatin^38^, fluorouracil^34^, and estradiol^39^.

## Discussion

We developed an approach to identify and validate drug repurposing candidates, which integrates disease gene expression signatures, drug perturbation data, and clinical data. For both hyperlipidemia and hypertension, we replicated known FDA-approved drugs and identified existing drugs approved for other diseases that had statistically significant biomarker-lowering effects. A substantial number of these biomarker-lowering effects are supported by evidence from multiple databases, the literature, and domain-expert review. Finally, we externally replicated the clinical validation pipeline in the NIH All of Us Research Program database, in which we observed similar drug treatment effect sizes.

While statistically significant, the biomarker-lowering effects associated with repurposing candidate exposure are not clinically significant. The drug repurposing candidates should not be used in place of known approved drugs for treating hyperlipidemia and hypertension (Table 2 and Supplementary Tables 1 and 2). As expected, known approved drugs had much larger therapeutic effect sizes compared to drugs approved for other diseases. For instance, individuals exposed to simvastatin (a known lipid-lowering drug) experienced much larger reductions in LDL-C compared to individuals exposed to valproate (−30.49 mg dL^−1^ vs. −4.71 mg dL^−1^) (Fig. 3a and Supplementary Data 13). Rather, this study’s contribution is a proof-of-concept approach to identify and clinically validate drug repurposing candidates. While hyperlipidemia and hypertension have many safe and potent drugs, there are still human diseases without effective treatments. For many of these diseases, our approach has the potential to identify existing drugs that may be more effective than current therapies. For these challenging diseases, gene expression signatures can be computed with S-PrediXcan using GWAS summary statistics that are publicly available in the GWAS catalog^43^ and UK Biobank^25^. At the time of writing, there are GWAS summary statistics for 869 and 7221 unique human conditions in the GWAS catalog and UK Biobank, respectively.

Compared to existing methods to validate repurposing candidates, our approach’s first advantage is the ability to measure drug efficacy in humans at scale. Similar to previous studies^16,17^, our approach allowed us to test many drugs (84 and 94 for hyperlipidemia and hypertension, respectively) in human individuals, because it uses automated informatics software to extract, process, and analyze EHR data. The ability to measure the magnitude of treatment effect is important for designing clinical trials, as lack of efficacy commonly causes clinical trials to fail^44^. In addition, testing many candidates enabled us to detect both potential true- and false-positive repurposing candidates. An example of a false-positive is sorafenib for treating hypertension. Sorafenib, a drug indicated for hepatocellular carcinoma, was predicted to lower SBP, because its iLINCS perturbation data reversed the hypertension gene expression signature (Supplementary Data 7). In the VUMC SD study, however, sorafenib increased SBP (5.12 mm Hg, *P* = 0.04; Fig. 3b and Supplementary Data 13), a side effect that has been previously reported^34^. In contrast to our approach, using more common validation strategies, like animal models and in vitro assays^9,10^, to test a similar number of drugs would have been cost- and time-prohibitive.

Our approach’s second advantage is its ease of portability. Previous studies have developed approaches to validate repurposing candidates using EHR data^16,17^. However, replicating drug repurposing signals from one database in a second independent database is often labor- and time-intensive, requiring many changes to the analysis pipeline due to institution-specific models used for storing clinical data^45^. In contrast, we replicated the VUMC SD pipeline with minor changes (essentially just changes to database table names) in the All of Us database, in under one week. This fast replication was possible because both databases store clinical data using the same standardized format^20^, the Observational Medical Outcomes Partnership (OMOP) Common Data Model (CDM)^46^.

Our approach’s last advantages are its ease of reproducibility and adaptability. Our analysis can be reproduced by other researchers because we used data from publicly available resources. The one exception are the individual-level clinical data stored in the VUMC SD. Importantly, researchers can easily reproduce the clinical validation studies in All of Us, because it uses a cloud computing infrastructure with data version control^47^. For their drug repurposing studies, researchers can adapt the software tools and computational notebooks that we have made publicly available (https://pwatrick.github.io/DrugRepurposingToolKit/).

Like other studies using observational clinical data, our approach has several limitations. Using observational data to measure treatment effects is challenging due to potential bias and confounding. In the clinical validation studies, we were particularly concerned about potential confounding by indication resulting in false-positive findings, i.e., a drug observed to reduce LDL-C does not truly reduce LDL-C. For instance, since individuals exposed to valproate experienced a statistically significant reduction in LDL-C (Fig. 3a), we infer that valproate lowers LDL-C. Another potential explanation for the LDL-C reduction is that many individuals taking valproate were also taking known lipid-lowering drugs, like statins. Recognizing this potential systematic error apriori, we excluded all individuals exposed to known lipid-lowering drugs during the observation period to reduce the risk of confounding by indication (Fig. 2b).

Another limitation shared by EHR-based studies is the fidelity of drug exposure data. Studies have shown that ~30–60% of individuals do not take preventative medications as prescribed^48^. One potential impact of this medication non-adherence is an underestimation of drug efficacy. However, we are encouraged by the replication of known drug effects in both databases that is consistent with efficacy rates reported in the literature. EHR-based studies can also be limited by information leakage, which may occur when individuals seek care from multiple providers who are not part of the study’s EHR system. For individuals whose medical records are fragmented, we do not have a completely accurate view of the individual’s health journey^49^. We reduce the effects of information leakage by requiring at least two outpatient visits with lab measurements within a span of 2 years.

In observational studies, another factor that can bias treatment effect estimates is that individuals are not randomly allocated to treatment groups, a common study design used in randomized clinical trials. To adjust for this potential bias, we used an SCCS study design (Fig. 2a), where individuals serve as their own controls, as it is believed to be robust to confounding^29^. We were able to use the SCCS design, as the two biomarkers we chose had efficacy measures (i.e., LDL-C and SBP measurements) that would be expected to occur soon after drug exposure. When future users apply our approach to validate repurposing candidates for diseases with delayed clinical endpoints (e.g., cancer and myocardial infarction), other approaches such as a retrospective cohort design may be more appropriate (Supplementary Table 3).

Looking forward, larger datasets from more diverse populations^50^ would enable researchers to uncover potential ancestry-selective drug effects. In this study, both the S-PrediXcan models and GWAS summary statistics were from cohorts composed primarily of European ancestry individuals. As a result, we may have missed drug repurposing candidates that would be effective in individuals of non-European ancestry. When genomic and clinical data from more diverse populations are made publicly available, our approach to identifying and validating drug repurposing candidates may improve. In the future, our approach can potentially be used to validate drug repurposing candidates for diseases with no effective treatments, like Alzheimer’s disease. In fact, while this manuscript was under review, a study was published that used EHRs to validate one drug repurposing candidate, bumetanide, for treating *APOE4*-related Alzheimer’s disease^51^.

In summary, we developed a high-throughput approach to identify drug repurposing candidates using gene expression signatures and to validate candidates using clinical EHR data. Our results suggest that the increasing amount of publicly available molecular and clinical data can be leveraged for drug repurposing studies.

## Methods

This study was conducted under all relevant ethical regulations with approval from the Vanderbilt University Medical Center Institutional Review Board (#180455) under a waiver of informed consent. Patients were not directly contacted for the study.

### Computation of disease gene expression signatures

We used disease gene expression signatures to represent the molecular state for the two diseases of interest, hyperlipidemia and hypertension (step 1 in Fig. 1a). Disease gene expression signatures were computed using the differentially expressed genes (DEGs) from individuals with the disease of interest compared to individuals without. To compute disease gene expression signatures, we used publicly available gene expression data^52,53^ imputed by S-PrediXcan;^11,12^ this method imputes genome-wide DEGs for a disease of interest using GWAS summary statistics for the disease of interest. S-PrediXcan was trained using data from the Genotype-Tissue Expression (GTEx) project^54^, which contains genotypes linked to RNA-seq data for 49 human tissues.

For hyperlipidemia, we computed the disease gene expression signatures using DEGs imputed using the whole blood elastic net model (Column “tissue” = “TW_Whole_Blood_Elastic_Net_0.5”)^55^ and GWAS summary statistics from the Global Lipids Genetics Consortium with 188,577 European ancestry individuals (Column “phenotype” = “GLGC_Mc_LDL”)^24^. We downloaded the hyperlipidemia DEGs file from “https://s3.amazonaws.com/imlab-open/Data/MetaXcan/results/metaxcan_results_database_v0.1.tar.gz”. For hypertension, we computed the disease gene expression signatures using DEGs imputed using an aggregate tissue model and GWAS summary statistics from a UK Biobank study with 340,159 European ancestry individuals (Column “phenotype” = “Systolic blood pressure, automated reading”)^25,56^. We downloaded the hypertension DEGs file (“smultixcan_4080_raw_ccn30.tsv.gz”) from “https://uchicago.box.com/shared/static/vket4ickq7qt3sj8dy3mv8zsr1our3xd.gz”.

Previous studies used various approaches to compute disease gene expression signatures^57^, and two of these approaches were used in this study. The first approach employed the widely used false discovery rate (FDR) metric with a cutoff of *q* < 0.05^58^ to compute the gene expression signatures for hyperlipidemia (Supplementary Data 3) and hypertension (Supplementary Data 5). The second approach was motivated by the algorithm used in So et al.^13^, as this was the first study to use S-PrediXcan imputed gene expression data to identify drug repurposing candidates. So et al. computed^13^ disease gene expression signatures using the *K*-most up- or downregulated genes, with *K* = 50, 100, 250, and 500. We selected the lower bound (*K* = 50), as we assumed that around 100 genes were sufficient to represent the molecular states for our diseases of interest. For hyperlipidemia, we ranked genes (by *Z* scores) from the most upregulated to the most downregulated genes. From this sorted list of DEGs, we computed the *K* = 50 hyperlipidemia gene expression signature by selecting the top fifty most up- and downregulated genes, for a total of 100 genes (Supplementary Data 2). For hypertension, we used expression values for genes that overlapped with those in the file, “suppl_table_S1-significant_gene_trait_associations.xlsx” (Column “trait” = “4080_raw-Systolic_blood_pressure_automated_reading”)^52^. The selected genes were predicted to be the most likely causal genes for SBP variation^52^. From this gene list, we computed the *K* = 50 hypertension gene expression signature, which was composed of 53 upregulated and 48 downregulated genes, for a total of 101 genes (Supplementary Data 4).

### Validation of disease gene expression signatures

To evaluate the robustness of the disease gene expression signatures, we queried the Drug-Gene Interaction Database (DGIdb)^59^. The DGIdb query allowed us to examine whether the disease-associated gene expression changes predicted by S-PrediXcan agreed with apriori expectations. For example, we expected apriori that in hyperlipidemia’s gene expression signature, *PCSK9*^26^ would be upregulated and *LDLR*^27^ would be downregulated.

### Using gene expression to find drug repurposing candidates

Next, we searched for drugs that reversed the S-PrediXcan imputed disease gene expression signatures (step 2 in Fig. 1a). To accomplish this, we queried the iLINCS database^18^. iLINCS hosts gene expression data from drug perturbation experiments. These in vitro experiments use a variety of cell types including human cancer cell lines^19^ and primary rat hepatocytes^60^. At the time of the study, iLINCS contained expression measurements for 74,201 genes from perturbation experiments of 21,299 small molecules^18^.

For both hyperlipidemia and hypertension, we uploaded their disease gene expression signatures to the iLINCS web portal. We used the default parameters in iLINCS to identify promising drug repurposing candidates. We matched disease and drug-gene expression signatures using either a weighted Pearson correlation^18^ or moderated *Z* scores^19^. Promising drug repurposing candidates were those with perturbations that reversed the S-PrediXcan imputed disease gene expression signature (i.e., had a negative correlation coefficient or concordance value) with a *P* < 0.05 for hyperlipidemia and *P* < 0.001 for hypertension (Fig. 1b).

For hyperlipidemia, we obtained drug repurposing candidates from the DrugMatrix dataset. DrugMatrix contains DEGs values for ~13,000 genes^60^. We used this set of drugs for hyperlipidemia because it contained data from primary liver tissue, a major tissue for regulating LDL-C levels. For hypertension, we obtained drug repurposing candidates from the Library of Integrated Network-based Cellular Signatures (LINCS) chemical perturbagen experiments. The LINCS dataset contains drug-gene expression signatures from the L1000 project^19^, derived mainly from in vitro human cancer cell line experiments. For hypertension, we selected drugs from the LINCS data and not from DrugMatrix (as was done for hyperlipidemia), because the top-ranked drugs in DrugMatrix were identified using data from perturbation experiments that used tissues not known to be major participants in regulating blood pressure.

Both hyperlipidemia and hypertension had two lists of drug repurposing candidates, one list generated using the *K* = 50 gene expression signature and another generated using the FDR gene expression signature. The lists were combined to create one iLINCS drug repurposing candidate list for each disease (Supplementary Data 6–7).

### Selecting drug candidates for clinical validation studies

From the iLINCS lists, we first mapped drug repurposing candidates to their bioactive ingredients in RxNorm. RxNorm is a standardized terminology linking drugs to concepts, which are unique terms that represent therapeutically equivalent medications^61^. Second, we excluded non-prescription drugs using the RxNorm CVF flag, 4096. Third, we excluded drugs with <20 individuals in the final cohort, both to ensure individual privacy (in the reporting of individual demographics) and for inadequate statistical power concerns (Supplementary Fig. 2).

### Identifying known FDA-approved drugs for target diseases

To identify known FDA-approved drugs for hypertension and hyperlipidemia, we used the MEDication Indication high-precision subset (MEDI-HPS) knowledge base^62^. MEDI-HPS links drug ingredients to diseases represented as International Classification of Diseases, Ninth Revision, Clinical Modification (ICD-9-CM) codes. To identify drug ingredients approved for treating hyperlipidemia, we used ICD-9-CM codes 272.0 “Pure hypercholesterolemia”, 272.2 “Mixed hyperlipidemia”, and 272.4 “Other and unspecified hyperlipidemia”. To identify drug ingredients approved for treating hypertension, we used the ICD-9-CM code, 401.9 “Hypertension NOS”. We then manually reviewed the drug lists and added drugs that were approved after MEDI-HPS was released (e.g., PCSK9 antibodies for hyperlipidemia).

### Clinical validation: EHR database and cohort description

To validate the drug repurposing candidates identified by gene expression signature matching, we quantified their efficacy on treating the diseases of interest; the clinical validation studies were conducted in the VUMC SD^28^, a de-identified copy of VUMC’s EHR (step 3 in Fig. 1a). The SD has longitudinal clinical data for >3.2 million individuals including billing codes, lab values, and medication exposure information. The SD is organized using the OMOP CDM^46^. For this study, VUMC SD data between 1995 and 2021 were used.

We validated drug repurposing candidates using clinical EHR data with an SCCS study design^29^ (Fig. 2a). Using SCCS allowed us to reduce the potential for false-positive therapeutic effects due to confounder bias. We designed the SCCS study by creating an observation window with two periods: baseline and treatment. The index date was the first date of exposure to the drug repurposing candidate of interest. The baseline period started before the index date and ended on the index date, with a maximum length of one year. The treatment period began after the index date and ended on the last date of exposure to the drug repurposing candidate, with a minimum length of thirty days (an induction period) and a maximum length of 1 year.

For each drug repurposing candidate, we identified a cohort of adults (≥18 years and <90 years) who were exposed to the drug repurposing candidate in the outpatient setting (Fig. 2b). Individuals were excluded if they did not have one or more outpatient biomarker measurements for the disease of interest, during both baseline and treatment periods. Individuals were also excluded if they were exposed to known FDA-approved drugs for the disease of interest. However, if the drug repurposing candidate being tested was a known FDA-approved drug for the disease of interest, then individuals were kept in the final cohort if they were solely excluded due to exposure to the drug repurposing candidate being tested. For instance, individuals exposed to simvastatin (a known lipid-lowering drug) were excluded in the analysis to clinically validate valproate as a drug repurposing candidate for hyperlipidemia; however, the same simvastatin-exposed individuals were not excluded in the study to validate simvastatin as a drug repurposing candidate for hyperlipidemia.

For the clinical validation studies, we report demographic statistics stratified by drug repurposing candidates. For gender and ethnicity, reported statistics are counts and percent of subgroups. We suppressed values if there were less than twenty individuals in the subgroup due to individual privacy concerns (Supplementary Data 9). For age and Elixhauser comorbidity index^63,64^, reported statistics are median and interquartile range (IQR) in the baseline and treatment periods; *P* values are from Wilcoxon signed-rank tests to identify statistically significant differences between baseline and treatment periods, with *P* < 0.05 considered statistically significant (Supplementary Data 10). For each Elixhauser comorbidity, reported are the number of individuals and percent of cohort with the comorbidity of interest in the baseline and treatment periods; *P* values are from McNemar’s tests to identify statistically significant differences between baseline and treatment periods, with *P* < 0.05 considered statistically significant. Elixhauser comorbidity counts were computed using ICD-9-CM and/or ICD-10-CM codes extracted from the start of the observation period to the end of baseline and treatment periods, respectively. We removed Elixhauser comorbidity statistics if there were less than twenty individuals in the subgroup due to individual privacy concerns (Supplementary Data 11).

### Clinical validation: biomarkers and drug efficacy

For hyperlipidemia, we clinically validated drug repurposing candidates using LDL-C as the biomarker. For the hypertension clinical validation study, we selected SBP as the biomarker. We chose to use LDL-C and SBP because they are measurements commonly collected for tracking disease progression and are important for predicting the risk of cardiovascular disease^65^. Further, we only used biomarker measurements taken in the outpatient setting, as inpatient biomarkers can be substantially altered by the acute disease processes related to inpatient admissions, and these altered biomarker measurements can confound the results of the clinical validation study.

We defined a drug repurposing candidate’s efficacy as the difference in median biomarker measurements taken before (baseline period) and after drug exposure (treatment period) (Fig. 2a). A repurposing candidate’s efficacy value was adjusted for confounding factors (see explanation of linear mixed model in the next section). We only used treatment period biomarker measurements taken after a 30-day induction period to allow each repurposing candidate to reach steady-state drug concentration. We removed median biomarker measurement outliers (defined as 1.5x interquartile range, outside the first and third quartiles) prior to statistical analysis. We used the magnitude of biomarker reduction to quantify a drug’s efficacy. For instance, in the hyperlipidemia study, drug A was more effective than drug B, if drug A-exposed individuals experienced larger reductions in LDL-C compared to drug B-exposed individuals.

### Clinical validation: statistical analysis

In the clinical validation studies, the null hypothesis was that individuals exposed to the drug repurposing candidate did not experience changes in the biomarker between the baseline and treatment periods. The alternative hypothesis was that individuals exposed to the drug repurposing candidate experienced changes in their biomarkers between the baseline and treatment periods. For each biomarker, we report the mean and SD of the median measurements during both baseline and treatment periods (Supplementary Data 12).

To determine whether individuals exposed to a drug repurposing candidate experienced significant biomarker changes, we used a linear mixed model^66,67^. For each drug repurposing candidate, we report the treatment effect as a point estimate (i.e., mean difference between median biomarker measurements from the baseline and treatment periods) with 95% CI and associated *P-*value from the linear mixed model (Supplementary Data 13). The treatment effect estimates were adjusted for age, gender, ethnicity, and disease comorbidity as seen in the following linear mixed model equation:

1 $$\begin{array}{c}\,{{{\mbox{biomarkerVal}}}}\sim {\beta }_{0}+{\beta }_{1}{{{\mbox{drugExposure}}}}+{\beta }_{2}{{{\mbox{Age}}}}+{\beta }_{3}{{{\mbox{Gender}}}}+{\beta }_{4}{{{\mbox{Ethnicity}}}}\\ +\,{\beta }_{5}{{{\mbox{Comorbidity}}}}+(1|{{{\mbox{Individual}}}})\end{array}$$

In this model, the variables drugExposure, Age, Gender, Ethnicity, and Comorbidity are treated as fixed effects, with a random intercept for each individual. In this paired study, each individual appears twice, once for the baseline period and a second time for the treatment period. During the baseline period, the continuous response variable, biomarkerVal, is the median of the biomarker measurements collected during the baseline period; the binary variable, drugExposure is set to “0” indicating that the individual was not exposed to the drug; the continuous variable Age is the individual’s normalized age at the end of the baseline period; the binary variable Gender is set to “1” if the individual is female and “0” otherwise; the binary variable Ethnicity is set to “1” if the individual is not white and “0” otherwise; the continuous variable Comorbidity is the individual’s normalized Elixhauser comorbidity index computed using ICD-9-CM and/or ICD-10-CM codes entered in the individual’s medical record beginning from the start of the observation period to the end of the baseline period.

During the treatment period, biomarkerVal is the median of the biomarker measurements collected during the treatment period; drugExposure is set to “1” indicating that the individual was exposed to the drug; Age is the individual’s age at the end of the treatment period; Gender and Ethnicity are equal to the individual’s baseline values (i.e., are time-invariant variables); Comorbidity is the individual’s normalized Elixhauser comorbidity index computed using ICD-9-CM and/or ICD-10-CM codes entered in the individual’s medical record beginning from the start of the observation period to the end of the treatment period. Each drug’s treatment effect estimate and the associated *P*-value is represented by *β*_1_.

A drug was deemed to have a statistically significant therapeutic effect if it had a negative point estimate (i.e., *β*_1_ < 0; exposure resulted in lower biomarker measurements in the treatment period, compared to baseline) with *P* < 0.05. We report both the number of drugs with *P* < 0.05 and the number of drugs with *P* values that crossed Bonferroni correction (0.05/84 = 5.95 × 10^-4^ for hyperlipidemia; 0.05/94 = 5.32 × 10^−4^ for hypertension) to adjust for multiple testing.

### External replication of clinical validation studies

To validate the findings from the VUMC SD, we performed external clinical validation studies using the NIH All of Us Research Program database^20,21^ (step 4 in Fig. 1a). The All of Us Research Program database is a unique resource with health data from a diverse group of participants, with >50% of participants as members of racial and ethnic minorities, and >80% from underrepresented groups in biomedical research. As of March 2021, the dataset contains >370,000 participants and EHRs for >236,000 participants with diverse backgrounds. Analyses were performed in the All of Us dataset v4, during the beta testing phase of the program, which began in May 2020^21^. For this study, All of Us data between 1991–2020 were used. We tested all drugs with statistically significant therapeutic effects (i.e., decreased LDL-C or SBP measurements at *P* < 0.05) in the VUMC SD clinical validation studies.

Results reported are in compliance with the All of Us Data and Statistics Dissemination Policy disallowing disclosure of group counts under 20 to protect participant privacy.

### Review of evidence to support novel repurposing candidates

We used multiple databases (SIDER^34^, DEB2^36^, and TWOSIDES^68^), the literature, and domain-expert review (S.N. and C.M.S.) to confirm the therapeutic effects in the VUMC SD clinical validation study, for drugs not FDA-approved for the diseases of interest. SIDER is a resource linking drugs to side effects, extracted from drug labels^34^. DEB2^36^ is a resource linking drugs to their indications and side effects; it was derived from five publicly available sources including SIDER, MEDLINE, and DrugBank. TWOSIDES is a resource containing statistics for potential drug-drug interactions derived from the FDA adverse event reporting system^68^.

### Reporting summary

Further information on research design is available in the Nature Research Reporting Summary linked to this article.

## Supplementary information

Supplementary Information

Reporting Summary

Description of Additional Supplementary Files

Supplementary Data 1

Supplementary Data 2

Supplementary Data 3

Supplementary Data 4

Supplementary Data 5

Supplementary Data 6

Supplementary Data 7

Supplementary Data 8

Supplementary Data 9

Supplementary Data 10

Supplementary Data 11

Supplementary Data 12

Supplementary Data 13

## Data Availability

The S-PrediXcan generated DEGs file for hyperlipidemia can be found at “https://s3.amazonaws.com/imlab-open/Data/MetaXcan/results/metaxcan_results_database_v0.1.tar.gz” and for hypertension can be found at “https://uchicago.box.com/shared/static/vket4ickq7qt3sj8dy3mv8zsr1our3xd.gz”. All requests for SD data are reviewed by Vanderbilt University Medical Center to determine whether the request is subject to any intellectual property or confidentiality obligations. Data are available through restricted access for approved studies and researchers who agree to conditions of use, such as but not limited to securely storing data and only using it for approved purposes. Any such data and materials that are approved will be released via a Data Use Agreement. The initial request can be sent to the corresponding author, and the applicants will be contacted within two weeks. De-identified data are available on the researcher workbench of the All of Us Research Program located at https://workbench.researchallofus.org. Our All of Us workspace can be shared to any All of Us researchers by contacting W-Q.W. Links for databases and datasets used in this study: iLINCS: http://www.ilincs.org/ilincs/; SIDER: http://sideeffects.embl.de/; DEB2: https://www.vumc.org/cpm/deb2; TWOSIDES: https://github.com/tatonetti-lab/nsides-release; DGIdb: https://www.dgidb.org/; MEDI-HPS: https://www.vumc.org/wei-lab/medi; All of Us: https://www.researchallofus.org/.

## Code availability

To obtain disease gene expression signatures, we used DEGs imputed using the MetaXcan python package (https://github.com/hakyimlab/MetaXcan). Hyperlipidemia disease gene expression signature was generated using S-PrediXcan from MetaXcan v0.5.0. Hypertension gene expression signature was generated using S-MultiXcan from MetaXcan v0.6.0.

Analyses were conducted using R version 4.0.5. R packages used were janitor_2.1.0, broom_0.7.9, vroom_1.5.4, forcats_0.5.1, stringr_1.4.0, dplyr_1.0.7, purrr_0.3.4, readr_2.0.0, tidyr_1.1.3, tibble_3.1.3, ggplot2_3.3.5, tidyverse_1.3.1, lubridate_1.7.10, glue_1.4.2, lme4_1.1-27.1, lmerTest_3.1-3, comorbidity_0.6.0.9000, ddiwas_0.1, and DrugRepurposingToolKit_0.2.1.

The software used to extract EHR data, data processing, and data analysis can be found at https://github.com/pwatrick/DrugRepurposingToolKit or 10.5281/zenodo.5747805. An example for matching disease and drug-gene expression signatures can be found at https://pwatrick.github.io/DrugRepurposingToolKit/articles/gene_expression_signature_matching_example.html. An example for performing a clinical validation study in the NIH All of Us Research Program database can be found at https://pwatrick.github.io/DrugRepurposingToolKit/articles/all_of_us_example.html. For data cleaning and processing, this package leverages datasets and functions from the *ddiwas*^69^ and *comorbidity*^70^ R packages.
