# Supplementary material for: Integrating gene expression and clinical data to identify drug repurposing candidates for hyperlipidemia and hypertension
Source: Nat Commun. 2022 Jan 10;13:46. doi: 10.1038/s41467-021-27751-1 (PMC8748496; doi:10.1038/s41467-021-27751-1)
Supplement: Supplementary file 1 — Supplementary Information [file 41467_2021_27751_MOESM1_ESM.pdf]

Integrating Gene Expression and Clinical Data to Identify Drug Repurposing Candidates for Hyperlipidemia and Hypertension

SUPPLEMENTARY INFORMATION

Supplementary Figures

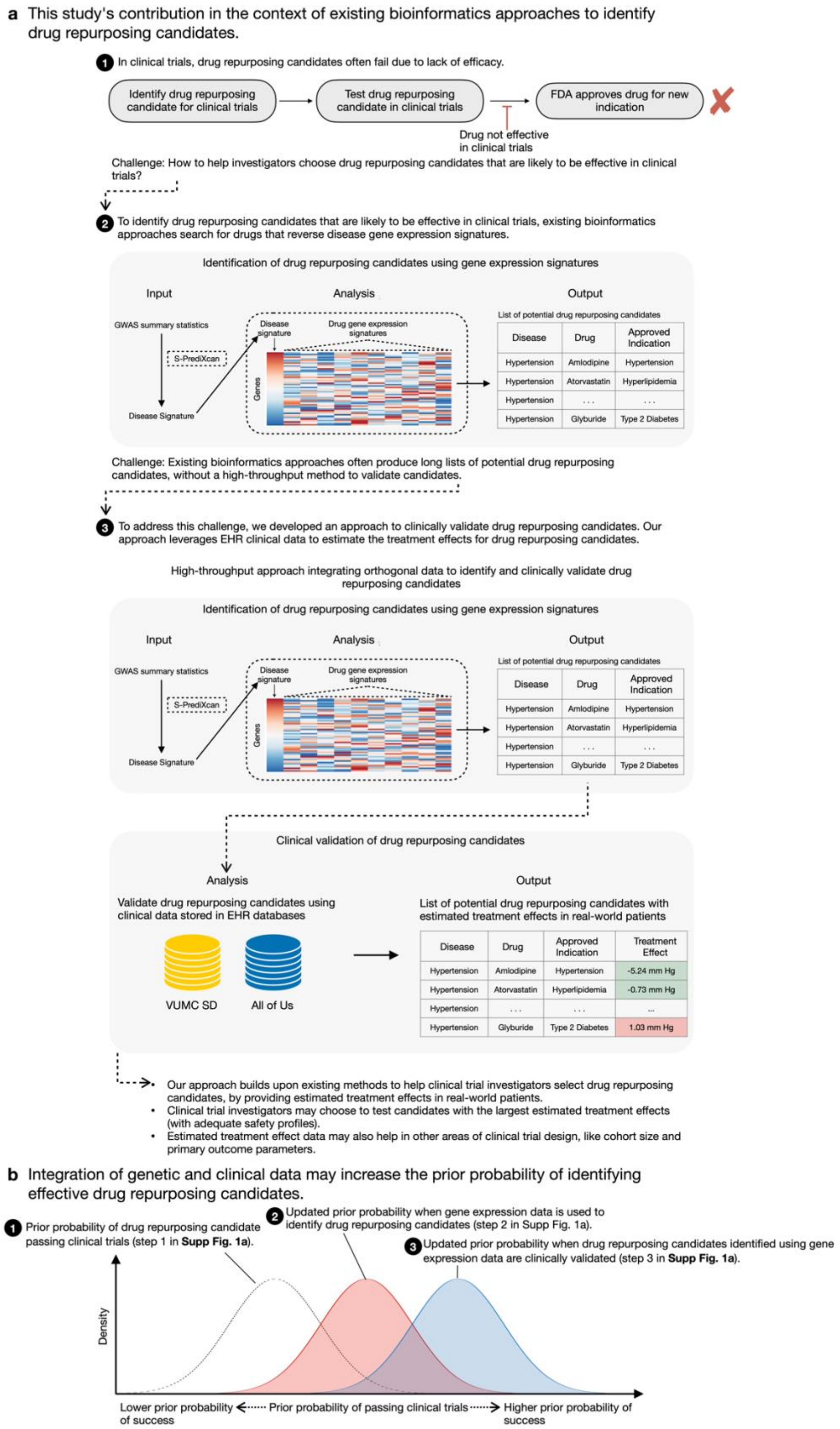

Supplementary Figure 1. **Overview of study's contributions to identifying drug repurposing candidates.** **a** Compared to existing bioinformatics approaches, our approach allows researchers to clinically validate drug repurposing candidates identified from gene expression data. **b** Conceptual framework for the value added by integrating genetic and clinical data to identify drug repurposing candidates for clinical trial testing. Note that this is just an illustration and a formal Bayesian-like analysis was not conducted.

Abbreviations. GWAS: genome-wide association study; EHR: electronic health record; VUMC: Vanderbilt University Medical Center; SD: Synthetic Derivativ

a

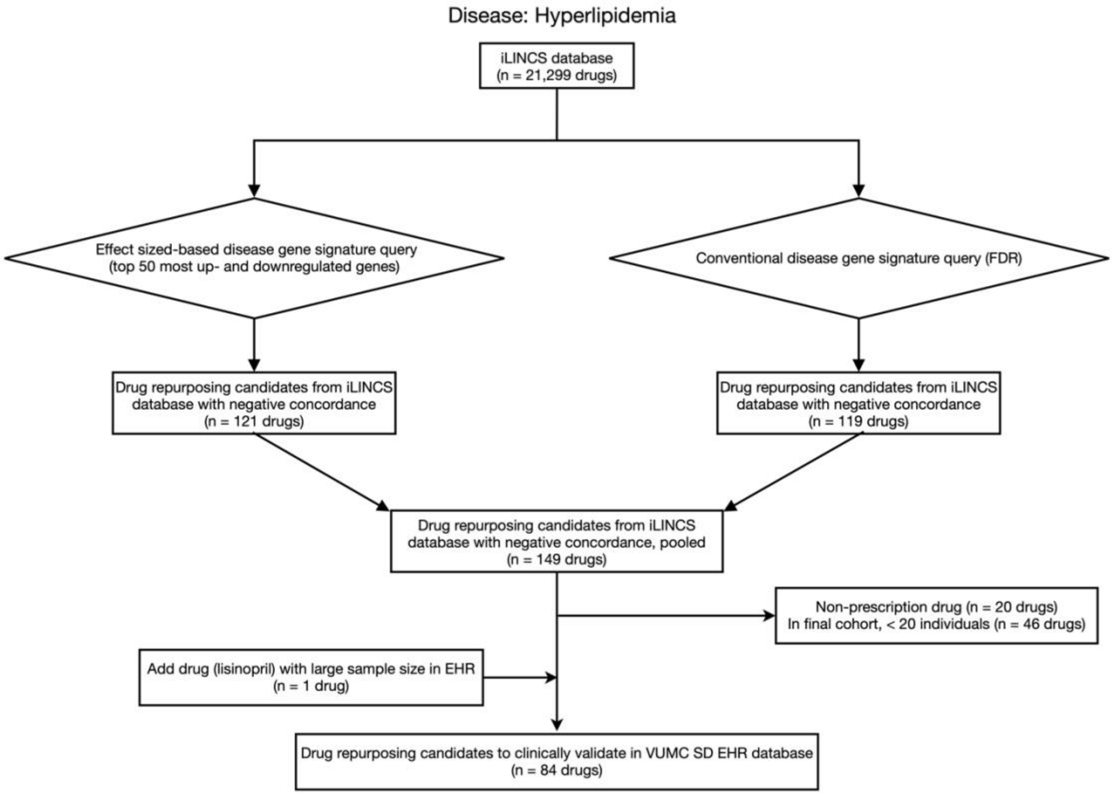
Disease: Hyperlipidemia
iLINCS database
(n = 21,299 drugs)
Effect sized-based disease gene signature query
(top 50 most up- and downregulated genes)
Conventional disease gene signature query (FDR)
Drug repurposing candidates from iLINCS
database with negative concordance
(n = 121 drugs)
Drug repurposing candidates from iLINCS
database with negative concordance
(n = 119 drugs)
Drug repurposing candidates from iLINCS
database with negative concordance, pooled
(n = 149 drugs)
Non-prescription drug (n = 20 drugs)
In final cohort, < 20 individuals (n = 46 drugs)
Add drug (lisinopril) with large sample size in EHR
(n = 1 drug)
Drug repurposing candidates to clinically validate in VUMC SD EHR database
(n = 84 drugs)

b

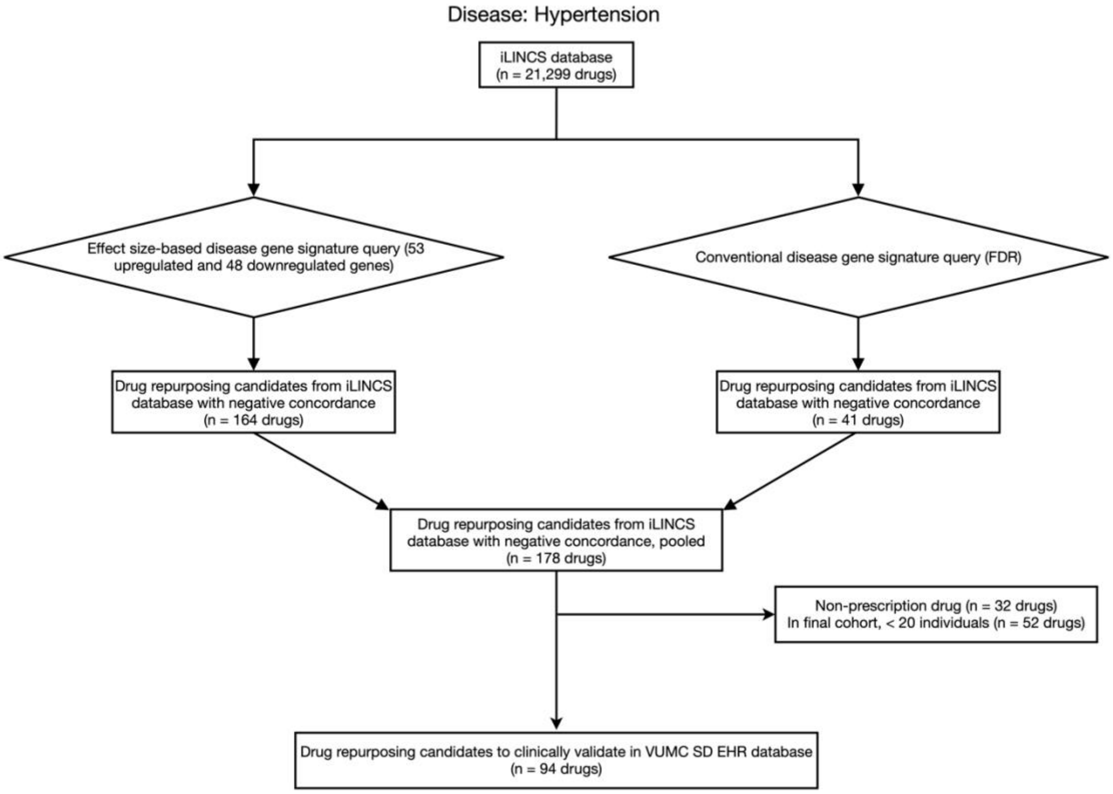
Disease: Hypertension
iLINCS database
(n = 21,299 drugs)
Effect size-based disease gene signature query (53
upregulated and 48 downregulated genes)
Conventional disease gene signature query (FDR)
Drug repurposing candidates from iLINCS
database with negative concordance
(n = 164 drugs)
Drug repurposing candidates from iLINCS
database with negative concordance
(n = 41 drugs)
Drug repurposing candidates from iLINCS
database with negative concordance, pooled
(n = 178 drugs)
Non-prescription drug (n = 32 drugs)
In final cohort, < 20 individuals (n = 52 drugs)
Drug repurposing candidates to clinically validate in VUMC SD EHR database
(n = 94 drugs)

Supplementary Figure 2. **Selection of drug repurposing candidates to clinically validate in VUMC SD.** **a** Selection of drug repurposing candidates from the iLINCS database to validate in the EHR, for hyperlipidemia. Lisinopril was added as a drug repurposing candidate because like quinapril, one of the drug repurposing candidates from iLINCS, lisinopril is an ACE inhibitor, but is much more commonly prescribed. **b** Selection of drug repurposing candidates from iLINCS database to validate in the EHR, for hypertension.

Abbreviations. VUMC: Vanderbilt University Medical Center; SD: Synthetic Derivative; EHR: electronic health record; iLINCS: Integrative Library of Integrated Network-Based Cellular Signatures; ACE: angiotensin-converting enzyme.

Supplementary Tables

Supplementary Table 1. Review of existing evidence to confirm the therapeutic effects for hyperlipidemia drug repurposing candidates observed in clinical validation studies.

Disease	Drug	Approved indication	Existing evidence supports therapeutic effect	Evidence description
Hyperlipidemia	Tamoxifen	Cancer	Yes	Breast cancer patients on tamoxifen experienced decreases in total serum cholesterol and LDL-C.[1]
Hyperlipidemia	Gentamicin	Bacterial infections	No	Most likely false-positive finding, as chronic drug exposure is uncommon. Also, the treatment effect was no longer statistically significant after removing non-systemic exposures.
Hyperlipidemia	Digoxin	Arrhythmias	Yes	Apolipoprotein E-deficient mice exposed to digoxin experienced reductions in LDL-C.[2]
Hyperlipidemia	Risperidone	Schizophrenia	No	Studies show that risperidone either has a negligible effect on or may increase LDL-C levels.[3]
Hyperlipidemia	Valproate	Seizure	Yes	Children with epilepsy on valproic acid had significantly lower mean LDL-C levels compared to healthy children.[4]
Hyperlipidemia	Methocarbamol	Muscle spasms	No	
Hyperlipidemia	Acetaminophen	Pain	No	

Abbreviations. RCT: randomized clinical trial; ACE: angiotensin-converting enzyme; LDL-C: low-density lipoprotein cholesterol; SSRIs: selective serotonin reuptake inhibitors.

Supplementary Table 2: Review of existing evidence to confirm the therapeutic effects for hypertension drug repurposing candidates observed in clinical validation studies.

Disease	Drug	Approved indication	Existing evidence supports therapeutic effect	Evidence description
Hypertension	Caffeine	Fatigue	No	Most likely false-positive finding, as caffeine is not commonly prescribed
Hypertension	Levofloxacin	Bacterial infections	Yes	Rapid intravenous infusion of levofloxacin can result in hypotension.[5]
Hypertension	Doxorubicin	Cancer	No	Most likely false-positive finding, as chronic drug exposure is uncommon.
Hypertension	Docetaxel	Cancer	Yes	SIDER: 2.8% of patients with solid tumors treated with docetaxel experienced hypotension.
Hypertension	Rosiglitazone	Type 2 Diabetes	Yes	In an RCT, patients randomized to metformin and rosiglitazone had lower blood pressures than patients randomized to the metformin and placebo group.[6]
Hypertension	Gemcitabine	Cancer	No	Most likely false-positive finding, as chronic drug exposure is uncommon.
Hypertension	Phenytoin	Seizure	Yes	DEB2: hypotension is a side effect of phenytoin.
Hypertension	Simvastatin	Hyperlipidemia	Yes	In an RCT, simvastatin was shown to lower blood pressure.[7]
Hypertension	Dexamethasone	Inflammation	No	SIDER: Hypotension can occur due to adrenal insufficiency, but hypertension seems more likely due to increased sodium levels, leading to edema.
Hypertension	Atorvastatin	Hyperlipidemia	Yes	In an RCT, atorvastatin was shown to lower blood pressure.[8]
Hypertension	Fluorouracil	Cancer	Yes	SIDER: 1% of patients exposed to fluorouracil experienced hypotension.
Hypertension	Escitalopram	Depression	No	SSRIs have been shown to increase blood pressure.[9]
Hypertension	Estradiol	Menopause	Yes	Estradiol exposure was shown to lower blood pressure.[10]
Hypertension	Sertraline	Depression	No	SSRIs have been shown to increase blood pressure.[9]
Hypertension	Ipratropium	Asthma	No	SIDER: hypotension and hypertension are both listed as potential side effects.
Hypertension	Celecoxib	Pain	No	SIDER: celecoxib can worsen hypertension.
Hypertension	Fluoxetine	Depression	No	SSRIs have been shown to increase blood pressure.[9]

Disease	Drug	Approved indication	Existing evidence supports therapeutic effect	Evidence description
Hypertension	Fexofenadine	Allergic Rhinitis	No	In an RCT, fexofenadine reduced vasodilation and postexercise hypotension.[11]

Abbreviations. RCT: randomized clinical trial; SSRIs: selective serotonin reuptake inhibitors.

Supplementary Table 3. Observational database study design catalog.

Expected time to clinical endpoint after exposure to drug	Study design	Clinical endpoint examples
Short (weeks-months)	SCCS	Fasting glucose/HbA1c levels[12,13]<br>Cholesterol levels[13]<br>Blood pressure[13]
Long (years)	Cohort	Coronary artery disease[14]<br>Cancer mortality[15,16]<br>Colectomy procedure[17]<br>Alzheimer's disease prevalence[18]

Abbreviations. SCCS: self-controlled case series; HbA1c: hemoglobin A1c

Supplementary References

1. Dnistrian, A. M., Schwartz, M. K., Greenberg, E. J., Smith, C. A. & Schwartz, D. C. Effect of tamoxifen on serum cholesterol and lipoproteins during chemohormonal therapy. *Clin. Chim. Acta* **223**, 43–52 (1993).

2. Shi, H. *et al.* Digoxin reduces atherosclerosis in apolipoprotein e-deficient mice. *Br. J. Pharmacol.* **173**, 1517–1528 (2016).

3. Newcomer, J. W. Second-Generation (atypical) antipsychotics and metabolic effects. *CNS Drugs* **19**, 1–93 (2005).

4. Eirís, J. M. *et al.* Effects of long-term treatment with antiepileptic drugs on serum lipid levels in children with epilepsy. *Neurology* **45**, 1155–1157 (1995).

5. *LEVOFLOXACIN injection [package insert]. Lake forest, IL: Akorn, inc*. (Akorn, Inc., 2020). https://dailymed.nlm.nih.gov/dailymed/medguide.cfm?setid=4438fed2-7ef5-488f-baa8-39bc65768d1d.

6. Negro, R., Mangieri, T., Dazzi, D., Pezzarossa, A. & Hassan, H. Rosiglitazone effects on blood pressure and metabolic parameters in nondipper diabetic patients. *Diabetes Res. Clin. Pract.* **70**, 20–25 (2005).

7. Correa, V., Jr *et al.* Blood pressure-lowering effect of simvastatin: A placebo-controlled randomized clinical trial with 24-h ambulatory blood pressure monitoring. *J. Hum. Hypertens.* **28**, 62–67 (2014).

8. Kanaki, A. I. *et al.* Low-dose atorvastatin reduces ambulatory blood pressure in patients with mild hypertension and hypercholesterolaemia: A double-blind, randomized, placebo-controlled study. *J. Hum. Hypertens.* **26**, 577–584 (2012).

9. Peixoto, M. F., Cesaretti, M., Hood, S. D. & Tavares, A. Effects of SSRI medication on heart rate and blood pressure in individuals with hypertension and depression. *Clin. Exp. Hypertens.* **41**, 428–433 (2019).

10. Seely, E. W., Walsh, B. W., Gerhard, M. D. & Williams, G. H. Estradiol with or without progesterone and ambulatory blood pressure in postmenopausal women. *Hypertension* **33**, 1190–1194 (1999).

11. Lockwood, J. M., Wilkins, B. W. & Halliwill, J. R. H1 receptor-mediated vasodilatation contributes to postexercise hypotension. *J. Physiol.* **563**, 633–642 (2005).

12. Brown, A. S., Rasooly, D. & Patel, C. J. Leveraging Population-Based clinical quantitative phenotyping for drug repositioning. *CPT Pharmacometrics Syst Pharmacol* **7**, 124–129 (2018).

13. Kim, D.-H. *et al.* High-Throughput algorithm for discovering new drug indications by utilizing Large-Scale electronic medical record data. *Clin. Pharmacol. Ther.* **108**, 1299–1307 (2020).

14. Cheng, F. *et al.* Network-based approach to prediction and population-based validation of in silico drug repurposing. *Nat. Commun.* **9**, 2691 (2018).

15. Wu, Y. *et al.* Discovery of noncancer drug effects on survival in electronic health records of patients with cancer: A new paradigm for drug repurposing. *JCO Clin Cancer Inform* **3**, 1–9 (2019).

16. Xu, H. *et al.* Validating drug repurposing signals using electronic health records: A case study of metformin associated with reduced cancer mortality. *J. Am. Med. Inform. Assoc.* **22**, 179–191 (2015).

17. Bai, L. *et al.* Computational drug repositioning of atorvastatin for ulcerative colitis. *J. Am. Med. Inform. Assoc.* (2021) doi:10.1093/jamia/ocab165.

18. Taubes, A. *et al.* Experimental and real-world evidence supporting the computational repurposing of bumetanide for APOE4-related alzheimer's disease. *Nature Aging* **1**, 932–947 (2021).
