# Supplementary material for: Integrating gene expression and clinical data to identify drug repurposing candidates for hyperlipidemia and hypertension
Source: Nat Commun. 2022 Jan 10;13:46. doi: 10.1038/s41467-021-27751-1 (PMC8748496; doi:10.1038/s41467-021-27751-1)
Supplement: Supplementary file 2 — Reporting Summary [file 41467_2021_27751_MOESM2_ESM.pdf]

nature portfolio

Corresponding author(s): Wei-Qi Wei

Last updated by author(s): Dec 8, 2021

Reporting Summary

Nature Portfolio wishes to improve the reproducibility of the work that we publish. This form provides structure for consistency and transparency in reporting. For further information on Nature Portfolio policies, see our Editorial Policies and the Editorial Policy Checklist.

Statistics

For all statistical analyses, confirm that the following items are present in the figure legend, table legend, main text, or Methods section.

n/a	Confirmed	
☐	☒	The exact sample size (*n*) for each experimental group/condition, given as a discrete number and unit of measurement
☐	☒	A statement on whether measurements were taken from distinct samples or whether the same sample was measured repeatedly
☐	☒	The statistical test(s) used AND whether they are one- or two-sided<br>*Only common tests should be described solely by name; describe more complex techniques in the Methods section.*
☐	☒	A description of all covariates tested
☐	☒	A description of any assumptions or corrections, such as tests of normality and adjustment for multiple comparisons
☐	☒	A full description of the statistical parameters including central tendency (e.g. means) or other basic estimates (e.g. regression coefficient) AND variation (e.g. standard deviation) or associated estimates of uncertainty (e.g. confidence intervals)
☐	☒	For null hypothesis testing, the test statistic (e.g. *F*, *t*, *r*) with confidence intervals, effect sizes, degrees of freedom and *P* value noted<br>*Give P values as exact values whenever suitable.*
☒	☐	For Bayesian analysis, information on the choice of priors and Markov chain Monte Carlo settings
☒	☐	For hierarchical and complex designs, identification of the appropriate level for tests and full reporting of outcomes
☐	☒	Estimates of effect sizes (e.g. Cohen's *d*, Pearson's *r*), indicating how they were calculated

Our web collection on statistics for biologists contains articles on many of the points above.

Software and code

Policy information about availability of computer code

Data collection	To obtain disease gene expression signatures, we used DEGs imputed using the MetaXcan python package (https://github.com/hakyimlab/MetaXcan). Hyperlipidemia disease gene expression signature was generated using S-PrediXcan from MetaXcan v0.5.0. Hypertension gene expression signature was generated using S-MultiXcan from MetaXcan v0.6.0. See main manuscript's Methods section, "Computation of disease gene expression signatures". To identify an initial set of drug repurposing candidates, the iLINCS database was searched. See main manuscript's Methods section, "Matching disease and drug gene expression signatures to identify drug repurposing candidates". To extract de-identified EHR data from the VUMC SD, SQL code was used to query an IBM Netezza database. To extract de-identified EHR data from the NIH All of Us Research database, SQL code was used to query Google's BigQuery Cloud Data Warehouse.
Data analysis	Analyses were conducted using R version 4.0.5. R packages used were janitor_2.1.0, broom_0.7.9, vroom_1.5.4, forcats_0.5.1, stringr_1.4.0, dplyr_1.0.7, purrr_0.3.4, readr_2.0.0, tidyr_1.1.3, tibble_3.1.3, ggplot2_3.3.5, tidyverse_1.3.1, lubridate_1.7.10, glue_1.4.2, lme4_1.1-27.1, lmerTest_3.1-3, comorbidity_0.6.0.9000, ddiwas_0.1, and DrugRepurposingToolKit_0.2.1.<br><br>The software used to extract EHR data, data processing, and data analysis can be found at https://github.com/pwatrick/DrugRepurposingToolKit or https://doi.org/10.5281/zenodo.5747805. An example for matching disease and drug gene expression signatures can be found at https://pwatrick.github.io/DrugRepurposingToolKit/articles/gene_expression_signature_matching_example.html. An example for performing a clinical validation study in the NIH All of Us Research Program database can be found at https://pwatrick.github.io/DrugRepurposingToolKit/articles/all_of_us_example.html.

For manuscripts utilizing custom algorithms or software that are central to the research but not yet described in published literature, software must be made available to editors and reviewers. We strongly encourage code deposition in a community repository (e.g. GitHub). See the Nature Portfolio guidelines for submitting code & software for further information.

Data

Policy information about availability of data

All manuscripts must include a data availability statement. This statement should provide the following information, where applicable:

- Accession codes, unique identifiers, or web links for publicly available datasets
- A description of any restrictions on data availability
- For clinical datasets or third party data, please ensure that the statement adheres to our policy

The S-PrediXcan generated DEGs file for hyperlipidemia can be found at "https://s3.amazonaws.com/imlab-open/Data/MetaXcan/results/metaxcan_results_database_v0.1.tar.gz" and for hypertension can be found at "https://uchicago.box.com/shared/static/vket4ickq7qt3sj8dy3mv8zsr1our3xd.gz".

All requests for SD data are reviewed by Vanderbilt University Medical Center to determine whether the request is subject to any intellectual property or confidentiality obligations. Data are available through restricted access for approved studies and researchers who agree to conditions of use, such as but not limited to securely storing data and only using it for approved purposes. Any such data and materials that are approved will be released via a Data Use Agreement. The initial request can be sent to the corresponding author, and the applicants will be contacted within two weeks.

De-identified data are available on the researcher workbench of the All of Us Research Program located at https://workbench.researchallofus.org. Our All of Us workspace can be shared to any All of Us researchers by contacting W-Q.W..

Links for databases and datasets used in this study:
iLINCS: http://www.ilincs.org/ilincs/
SIDER: http://sideeffects.embl.de/
DEB2: https://www.vumc.org/cpm/deb2
TWOSIDES: https://github.com/tatonetti-lab/nsides-release
DGIdb: https://www.dgidb.org/
MEDI-HPS: https://www.vumc.org/wei-lab/medi
All of Us: https://www.researchallofus.org/.

Field-specific reporting

Please select the one below that is the best fit for your research. If you are not sure, read the appropriate sections before making your selection.

- [x] Life sciences
- [ ] Behavioural & social sciences
- [ ] Ecological, evolutionary & environmental sciences

For a reference copy of the document with all sections, see nature.com/documents/nr-reporting-summary-flat.pdf

Life sciences study design

All studies must disclose on these points even when the disclosure is negative.

Sample size	For clinical validation studies, all adult patients, between 18 and 90 years old, in both VUMC SD and NIH All of Us databases were included in the study.
Data exclusions	Data exclusion decisions were made prior to statistical analysis. All patients exposed to the drug repurposing candidates and biomarkers of interests were included, except those patients with outlier median biomarker measurements (defined as 1.5 x interquartile range, outside the first and third quartiles).
Replication	External replication of the clinical validation pipeline was performed in the NIH All of Us Research database v4. Some findings in the VUMC SD study were not replicated due to sample size limitations. See Figure 4.
Randomization	We did not perform blinding, because we used existing observational EHR data. Clinical validation studies were performed using a self-controlled case series (SCCS) study design. See Figure 2a.
Blinding	We did not perform blinding, because we used existing observational EHR data.

Reporting for specific materials, systems and methods

We require information from authors about some types of materials, experimental systems and methods used in many studies. Here, indicate whether each material, system or method listed is relevant to your study. If you are not sure if a list item applies to your research, read the appropriate section before selecting a response.

Materials & experimental systems

n/a	Involved in the study
[x]	[ ] Antibodies
[ ]	[x] Eukaryotic cell lines
[x]	[ ] Palaeontology and archaeology
[x]	[ ] Animals and other organisms
[x]	[ ] Human research participants
[x]	[ ] Clinical data
[x]	[ ] Dual use research of concern

Methods

n/a	Involved in the study
[x]	[ ] ChIP-seq
[x]	[ ] Flow cytometry
[x]	[ ] MRI-based neuroimaging

Eukaryotic cell lines

Policy information about cell lines

Cell line source(s)	Drug gene expression signatures were calculated from gene expression data hosted on the iLINCS platform. This study used gene expression data obtained from a range of human cell lines and primary animal tissue. See iLINCS paper, "Pilarczyk, M. et al. Connecting omics signatures of diseases, drugs, and mechanisms of actions with iLINCS. bioRxiv 826271 (2019) doi:10.1101/826271".
Authentication	See iLINCS paper, "Pilarczyk, M. et al. Connecting omics signatures of diseases, drugs, and mechanisms of actions with iLINCS. bioRxiv 826271 (2019) doi:10.1101/826271".
Mycoplasma contamination	See iLINCS paper, "Pilarczyk, M. et al. Connecting omics signatures of diseases, drugs, and mechanisms of actions with iLINCS. bioRxiv 826271 (2019) doi:10.1101/826271".
Commonly misidentified lines (See ICLAC register)	See iLINCS paper, "Pilarczyk, M. et al. Connecting omics signatures of diseases, drugs, and mechanisms of actions with iLINCS. bioRxiv 826271 (2019) doi:10.1101/826271".
