# Supplementary material for: Integrating gene expression and clinical data to identify drug repurposing candidates for hyperlipidemia and hypertension
Source: Nat Commun. 2022 Jan 10;13:46. doi: 10.1038/s41467-021-27751-1 (PMC8748496; doi:10.1038/s41467-021-27751-1)
Supplement: Supplementary file 3 — Description of Additional Supplementary Files [file 41467_2021_27751_MOESM3_ESM.pdf]

Description of Additional Supplementary Files

File Name: Supplementary Data 1
Description: **Known disease-associated genes found in disease gene expression signatures.** Columns are disease, gene, target_name, gene_expression_change, source, interaction_type, drug_name, and drug_type.

File Name: Supplementary Data 2
Description: **S-PrediXcan estimated transcriptomic signatures used to query iLINCS for hyperlipidemia drug repurposing candidates, K = 50.** Columns are disease, gene, zscore, and pval. *P* values are from two-tailed association (elastic net) tests between S-PrediXcan predicted gene expression variation and LDL-C levels.[1]
- Abbreviations. iLINCS: Integrative Library of Integrated Network-based Cellular Signatures; LDL-C: low-density lipoprotein cholesterol.

File Name: Supplementary Data 3
Description: **S-PrediXcan estimated transcriptomic signatures used to query iLINCS for hyperlipidemia drug repurposing candidates, FDR (q < 0.05).** Columns are disease, gene, zscore, pval, and qval. *P* values are from two-tailed association (elastic net) tests between S-PrediXcan predicted gene expression variation and LDL-C levels.[1]
- Abbreviations. iLINCS: Integrative Library of Integrated Network-based Cellular Signatures; LDL-C: low-density lipoprotein cholesterol.

File Name: Supplementary Data 4
Description: **S-PrediXcan estimated transcriptomic signatures used to query iLINCS for hypertension drug repurposing candidates, K = 50.** Columns are disease, gene, zscore, and pval. *P* values are from two-tailed association (elastic net) tests between S-MultiXcan predicted gene expression variation and SBP readings.[2]
- Abbreviations. iLINCS: Integrative Library of Integrated Network-based Cellular Signatures; SBP: systolic blood pressure.

File Name: Supplementary Data 5
Description: **S-PrediXcan estimated transcriptomic signatures used to query iLINCS for hypertension drug repurposing candidates, FDR (q < 0.05).** Columns are disease, gene, zscore, pval, and qval. *P* values are from two-tailed association (elastic net) tests between S-MultiXcan predicted gene expression variation and SBP readings.[2]
- Abbreviations. iLINCS: Integrative Library of Integrated Network-based Cellular Signatures; SBP: systolic blood pressure.

File Name: Supplementary Data 6
Description: **Aggregated iLINCS drug repurposing candidate list for hyperlipidemia.** Columns are disease, signatureid, drug, concentration, tissue, time, concordance, pval. *P* values are from two-tailed weighted Pearson correlation tests between S-PrediXcan predicted gene expression levels and drug-perturbation induced gene expression changes from iLINCS.[3]
- Abbreviations. iLINCS: Integrative Library of Integrated Network-based Cellular Signatures.

File Name: Supplementary Data 7
Description: **Aggregated iLINCS drug repurposing candidate list for hypertension.** Columns are disease, drug, correlation, zscore, pval. *P* values are from two-tailed Empirical Bayes weighted t-tests.[4]
- Abbreviations. iLINCS: Integrative Library of Integrated Network-based Cellular Signatures.

File Name: Supplementary Data 8
Description: **Cohort selection numbers.** Columns are:
"Source",
"Drug",
"Disease",
"Exposed to drug repurposing candidate in outpatient setting and
had ≥1 outpatient biomarker measurements within one year after index date",
"In the EHR, did not have evidence for continued exposure to drug repurposing candidate after 30 day induction period",
"At start of observation period, was not ≥ 18 y or < 90 y",
"Did not have ≥1 outpatient biomarker measurements during both baseline and treatment periods",
"Exposed to known FDA-approved drug for disease of interest (with exception if drug being tested is FDA-approved for disease of interest)", and
"Final clinical validation cohort".

File Name: Supplementary Data 9
Description: **Demographic characteristics of clinical validation study cohorts.** Columns are source, disease, drug, n, n_female, pct_female, n_white, pct_white, n_black, pct_black, obs_period_length_median, obs_period_length_iqr, treatment_period_length_median.

Column definitions:
- "source": Source of data, either Vanderbilt (i.e., VUMC SD) or All of Us.
- "disease": Target disease, either hyperlipidemia or hypertension.
- "drug": Name of drug tested in clinical validation study.
- "n": Total number of individuals in cohort.
- "n_female": Number of female individuals in cohort.
- "pct_female": Percentage of cohort who were female.
- "n_white": Number of white individuals in cohort.
- "pct_white": Percentage of cohort who were white.
- "n_black": Number of black individuals in cohort.
- "pct_black": Percentage of cohort who were black.
- "obs_period_length_median": Observation period length, days; median.
- "obs_period_length_iqr": Observation period length, days; interquartile range.
- "treatment_period_length_median": Treatment period length, days; median.
- "treatment_period_length_iqr": Treatment period length, days; interquartile range.
- "*": If there were less than twenty individuals in "n_female", "n_white", "n_black", then values in all three columns and their associated percentages were suppressed to protect individual privacy.

File Name: Supplementary Data 10
Description: **Age and Elixhauser indices for clinical validation study cohorts.** Columns are source, disease, drug, n, variable, baseline_median, baseline_iqr, treatment_median, treatment_iqr, and pval.

Column definitions:
- "source": Source of data, either Vanderbilt (i.e., VUMC SD) or All of Us.
- "disease": Target disease, either hyperlipidemia or hypertension.
- "drug": Name of drug tested in clinical validation study.

- "n": Total number of individuals in cohort.
- "variable": "Age, y" = age in years; "Elixhauser Score" = Elixhauser comorbidity index.
- "baseline_median": Median of variable during baseline period.
- "baseline_iqr": Interquartile range of variable during baseline period.
- "treatment_median": Median of variable during treatment period.
- "treatment_iqr": Interquartile range of variable during treatment period.
- "pval": Two-tailed *P* values were calculated using Wilcoxon signed rank tests to identify statistically significant differences between baseline and treatment periods.

File Name: Supplementary Data 11
Description: **Elixhauser comorbidity statistics.** Columns are source, disease, drug, n, comorbidity, baseline_count, baseline_pct, treatment_count, treatment_pct, pval.

Column definitions:
- "source": Source of data, either Vanderbilt (i.e., VUMC SD) or All of Us.
- "disease": Target disease, either hyperlipidemia or hypertension.
- "drug": Name of drug tested in clinical validation study.
- "n": Total number of individuals in cohort.
- "comorbidity": Elixhauser comorbidity.
- "baseline_count": Number of individuals in cohort with comorbidity during baseline period.
- "baseline_pct": Percentage of cohort with comorbidity during baseline period.
- "treatment_count": Number of individuals in cohort with comorbidity during treatment period.
- "treatment_pct": Percentage of cohort with comorbidity during treatment period.
- "pval": - Two-tailed *P* values were calculated using McNemar’s test.

- If there were less than twenty individuals in either "baseline_count" or "treatment_count" columns, then the statistics were not reported.

File Name: Supplementary Data 12
Description: **Baseline and treatment period biomarker measurements.** Columns are source, disease, drug, n, biomarker, baseline_biomarker_mean, baseline_biomarker_sd, treatment_biomarker_mean, and treatment_biomarker_sd.

Columns definitions:
- "source": Source of data, either Vanderbilt (i.e., VUMC SD) or All of Us.
- "disease": Target disease, either hyperlipidemia or hypertension.
- "drug": Name of drug tested in clinical validation study.
- "n": Total number of individuals in cohort.
- "biomarker": Identity of biomarker, either LDL Cholesterol or Systolic Blood Pressure.
- "baseline_biomarker_mean": Mean of biomarker for cohort during baseline period.
- "baseline_biomarker_sd": Standard deviation of biomarker for cohort during baseline period.
- "treatment_biomarker_mean": Mean of biomarker for cohort during treatment period.
- "treatment_biomarker_sd": Standard deviation of biomarker for cohort during treatment period.

File Name: Supplementary Data 13
Description: **Treatment effects of drug repurposing candidates from clinical validation studies.** Columns are source, disease, drug, biomarker, n, estimate, se, conf.low, conf.high, pval, bonf, sig_50, and sig_fdr.

Column definitions:
- "source": Source of data, either Vanderbilt (i.e., VUMC SD) or All of Us.
- "disease": Target disease, either hyperlipidemia or hypertension.
- "drug": Name of drug tested in clinical validation study.
- "biomarker": Identity of biomarker, either LDL Cholesterol or Systolic Blood Pressure.
- "n": Number of total individuals in cohort.

$$biomarkerVal \sim \beta_0 + \beta_1 drugExposure + \beta_2 Age + \beta_3 Gender + \beta_4 Ethnicity + \beta_5 Comorbidity + (1|Individual) \quad (1)$$

- "estimate": β_1 point estimate from linear mixed model, Equation (1).
- "se": Standard error of β_1 from linear mixed model, Equation (1).
- "conf.low" and "conf.high": 95% confidence interval of β_1 from linear mixed model, Equation (1).
- "pval": Two-tailed *P* value of β_1 from linear mixed model, Equation (1).
- "bonf": "yes" indicates that the point estimate < 0 with p-value crossing Bonferroni correction (hyperlipidemia = 0.05/84 = 5.95×10^{-4}; hypertension = 0.05/94 = 5.32×10^{-4}).
- "sig_50" and "sig_fdr": "yes" means that the drug was found using the K = 50 genes and commonly used FDR metric (q < 0.05) to generate disease gene signatures, and "no" means that the drug was not found using the respective approach.

References

1. Barbeira, A. N. *et al.* Exploring the phenotypic consequences of tissue specific gene expression variation inferred from GWAS summary statistics. *Nat. Commun.* **9**, 1825 (2018)

2. Pividori, M. *et al.* PhenomeXcan: Mapping the genome to the phenome through the transcriptome. *Sci Adv* **6**, (2020)

3. Engreitz, J. M. *et al.* Content-based microarray search using differential expression profiles. *BMC Bioinformatics* **11**, 603 (2010)

4. Pilarczyk, M. *et al.* Connecting omics signatures of diseases, drugs, and mechanisms of actions with iLINCS. *bioRxiv* 826271 (2019) doi:10.1101/826271
